# Heartbreakers: Decoding the Molecular Dynamics of Stimulants Abuse on the Cardiovascular System

**DOI:** 10.15190/d.2025.6

**Published:** 2025-05-10

**Authors:** Cristina Mihaela Stirbu, Anisia-Cristiana Vasiliniuc, Anda Vasiliu, Elisa Anamaria Liehn

**Affiliations:** ^1^Center of Innovation and e-Health, Carol Davila University of Medicine and Pharmacy, Strada Dionisie Lupu 37, 030167 București, Bucharest, Romania; ^2^Military Medicine Institute, 3-5 Institutul Medico-Militar Street, 010919 Bucharest, Romania; ^3^Genomics Research and Development Institute, Bucharest, Romania; ^4^National Heart Center Singapore, 5 Hospital Dr, 169609, Singapore; ^5^St. Katharinen Hospital Frechen, 1-5 Kapellenstrasse, 50226 Frechen, Germany

**Keywords:** Substance abuse; stimulants; cardiovascular disease; drug consumer

## Abstract

This comprehensive review explores the intricate relationship between stimulants abuse, particularly involving caffeine, amphetamines, methylphenidate and cocaine, offering a detailed and insightful overview of the multifaceted consequences of stimulant drug use on the cardiovascular system. Substance abuse is a medical and socioeconomic issue, which should be regarded properly with respect to its multifaceted implications. For instance, chronic usage exponentially increases the cardiovascular risk, leading to high morbidity and mortality rates. Arrhythmias, atherosclerosis, thrombosis and ischemia are just some of the direct effects of stimulants on vessels and heart, which lead in time to more severe pathological changes, such as hypertension, cardiomyopathies, valvulopathies, myocardial infarction, peripheral artery disease and other cerebrovascular diseases. The different approaches and diverse perspectives of this review, highlight the clinical significance of this complex field. Moreover, opportunities for further research and exploration are pointed out, thus  promoting public education, personalized medical approaches, and targeted interventions to mitigate the substantial morbidity and mortality linked to stimulant abuse. Hopefully, this effort will enhance public cardiovascular well-being.

## SUMMARY

1. *Introduction *

*2. Stimulants: the heartbreaker effects:*

- *2.1. Perturbing the beat: Arrhythmia *
- *2.2. Cardiac heavy lifts: Hypertension *
- *2.3. Pipe predicament: Atherosclerosis *
- *2.4. Blocking the flow: Thrombosis and Thromboembolism *
- *2.5. Oxygen strike: Ischemia and Myocardial Infarction *
- *2.6. Harmony disruption: Cardiomyopathy *
- *2.7. Confused wings: Valvulopathy *
- *2.8. Invasion unleashed: Vasculitis *
- *2.9. Brain maze: Cerebrovascular Disease *
- *2.10. Mindset makeover: Future Perspectives *

*3. Conclusion*

## 1. Introduction

Substance abuse represents an important economic burden for society, implying huge healthcare costs. Stimulants represent the majority of abused substances since they are used not only for recreational purposes, but also for increased sport and cognitive performance, being present in various commercialized supplements^1-3^. Stimulants abuse is the excessive and compulsive use of stimulants in a detrimental way to the individual, regarding all aspects of their life, personal, social and professional^4,5^.

By recommendation of mental health professionals^6^, substance abuse is categorized in the same class as addiction or alcoholism. Thus, the diagnosis of substance abuse, medically termed as „substance use disorder”, is a complex process based on multiple criteria which are present in certain individuals for more than 12 months. Similarly, substance abuse can be classified as mild, moderate or severe, depending on the number of criteria met^7,8^.

This narrative review is based on literature sourced from PubMed and Scopus databases, focusing on English-language abstracts and full-text articles that examine the cardiovascular effects of stimulant abuse. This review focuses on some of the most common and severe cardiovascular pathologies resulting from the abuse of stimulants, highlighting the importance of stopping this phenomenon from both a humanitarian and economic perspective, especially among the younger generation. While many stimulants exist, this review will explore the central nervous system stimulants with the widest usage, such as caffeine, amphetamines, methylphenidate and cocaine^1,3^. Although nicotine can also be a stimulant, its cardiovascular effects are widely known and extensively summarized in the literature^9-11^ and it is not the aim of the current review.

The need for this review is justified by contradictions in the recent literature regarding the side effects of stimulants, which happened especially in the case of caffeine. Additionally, underreporting is a significant issue and much of the existing knowledge is based on case reports and small studies. This means that many mechanisms and side effects are either unknown or hypothesized and not yet proven with statistical significance. This review aims to update the current understanding of stimulants and to highlight the problem of underreporting, advocating for further, larger studies in this field.

Central nervous system (CNS) stimulants, such as amphetamines, methylphenidate, and cocaine, primarily exert their effects through the enhancement of neurotransmitter activity in the brain, particularly by increasing the levels of dopamine, norepinephrine, and serotonin within the synaptic cleft^1,3^. These substances achieve this by either promoting the release of these neurotransmitters or inhibiting their reuptake, leading to heightened alertness, improved attention, and increased energy^1^. However, the full spectrum of their actions is not completely understood. Concurrently, CNS stimulants have significant cardiovascular effects, including increased heart rate, elevated blood pressure, and enhanced cardiac output, primarily due to the stimulatory effects on the sympathetic nervous system. These changes can lead to acute risks such as arrhythmias, myocardial infarction, and, with chronic use, can contribute to the development of cardiovascular diseases^1,3^. Despite extensive research, gaps remain in our understanding of the precise molecular mechanisms underlying these cardiovascular effects and the variability in individual responses, indicating the need for further studies to elucidate these complex interactions fully. Throughout this review, these deficiencies in our current knowledge will be highlighted, emphasizing the importance of continued investigation into the cardiovascular impacts of central nervous system stimulants.

Caffeine is a natural compound, found in plants^12^, widely used as a stimulant at a global level^13,14^. It is important to note that health professionals do not classify caffeine into the same category as other stimulants, since caffeine consumers do not fulfill the criteria of substance use disorder. Nevertheless, there is evidence of mild withdrawal symptoms in certain contexts^6^, and it was considered an important factor for hypertension, arrhythmia and increased incidence of cardiovascular events.

Amphetamines were developed as synthetic drugs, but naturally occurring amphetamines can be found in plants such as Catha edulis^1,3,15^. The main used amphetamines are currently amphetamine and methamphetamine^15^.

Methylphenidate is also a synthetic substance that is widely used for the treatment of Attention Deficit Hyperactivity Disorder^16^.

Cocaine is a natural substance, isolated from the leaves of the coca plant^3^. Initially, it was known for its positive health effects in low dosages and was included in numerous beverages such as Coca Cola. Currently, severe health damages, high addiction potential and increased mortality among consumers are well known, being included between the illegal substances in the majority of countries^1^.

Stimulants have a wide range of short-term and long-term adverse effects, depending on several factors, such as the user's body weight, the specific stimulant employed, the dosage administered, the concurrent use of other drugs and stimulants, oral intake on an empty stomach and the individual's tolerance.

The adverse effects associated with stimulant abuse include: decreased appetite, anxiety, jitteriness, headaches, weight loss, insomnia, psychosis, pruritus, paranoia, sweating, shortness of breath, seizures, dangerously elevated body temperature, increased cardiovascular risks from persistently accelerated heart rate, vasoconstriction, and increased blood pressure, hostility, violent behavior^17^.

Particularly, stimulants impairing the uptake of dopamine, norepinephrine, and serotonin, in the synaptic cleft, such as amphetamines and cocaine, have highly addictive potential due to their euphoric effect^7^.

## 2. Stimulants: the heartbreaker effects

Among all known adverse effects of these stimulants, the cardiovascular alterations are the most dangerous, being responsible for increased morbidity and mortality of drug consumers. The exact mechanisms of the implied cardiovascular pathologies are not well known. However, it is speculated that stimulants alter endothelial cells and induce channelopathies, thus leading to arrhythmia, myocardial infarction, cerebrovascular diseases, etc.^18-20^. 

**Figure 1:**
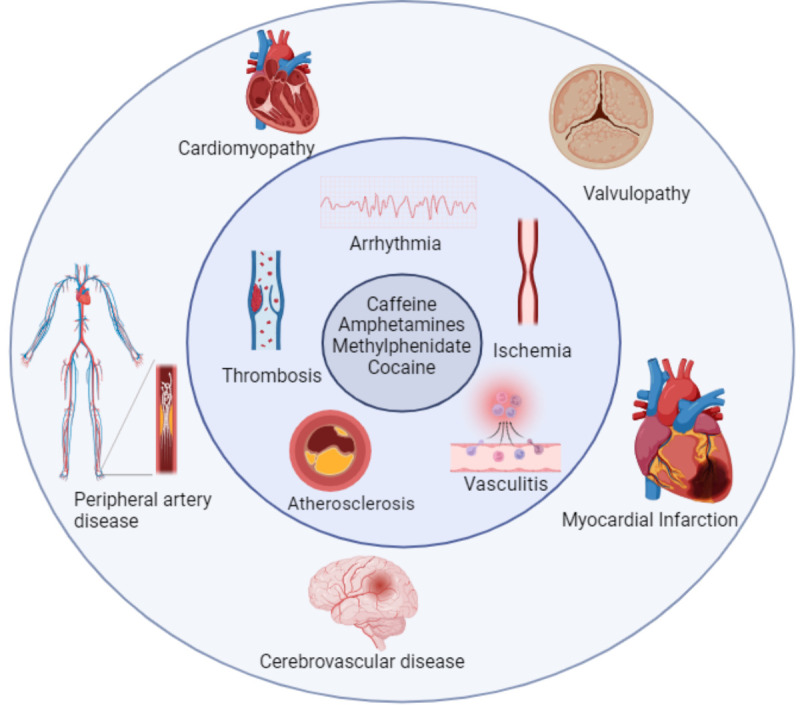
Primary and secondary cardiovascular effects following abuse of caffeine, amphetamines, methylphenidate, cocaine: arrhythmia, atherosclerosis, ischemia, vasculitis, thrombosis will lead in time to cardiomyopathy, valvulopathy, peripheral artery disease, myocardial infarction, cerebrovascular disease.

The cardiovascular pathologies induced by substance abuse are multifaceted, involving all components of the heart and vessels (Figure 1).

### 2.1. Perturbing the beat: Arrhythmia

The main and most dangerous cardiovascular complication of stimulant abuse is represented by arrhythmia. The arrhythmogenic effect of caffeine consumption is highlighted in several small studies^21-23^, particularly for individuals with associated comorbidities as well as with congenital cardiovascular malformations^24,25^. Caffeine intake stimulates cardiac RgR2 ryanodine receptors, inhibiting phosphodiesterase and thus having a cardio-stimulatory effect. This can imply arrhythmias, tachycardia, and in more severe situations, ventricular fibrillation^26^. Despite the conclusions of these smaller studies, a larger epidemiological analysis led to the exact opposite conclusion, as caffeine intake was actually found to significantly lower risk of arrhythmia (P < 0.0001 - for 4-5 cups/day of ground coffee and for 2-3 cups/day of instant coffee)^27,28^.

**Figure 2:**
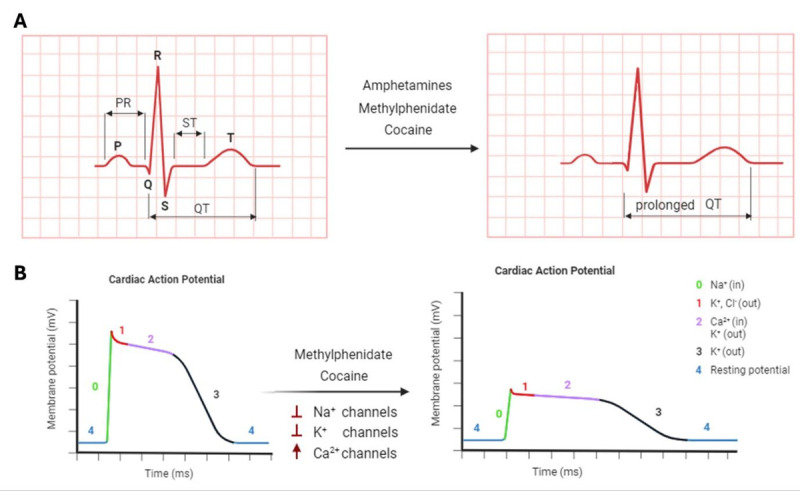
Mechanisms of arrhythmia induced by stimulants The electrocardiogram changes due to stimulant abuse. (B) Changes in cardiac action potential and ion channels activity during stimulant abuse.

Amphetamines are also known to be arrhythmogenic. For example, methamphetamine causes prolonged QT changes in electrocardiograms (Figure 2)^29^, increasing the risk for ventricular tachycardia^30^. The arrhythmogenic effect can be aggravated by existent fibrosis in the myocardium^31,32^. Methylphenidate administration, particularly in children with attention deficit hyperactivity disorder, is known to increase heart rate, with effects observed in animal models^33^, and even in clinical trials^34-37^. As a mechanism, the prolongation of QT intervals was observed in most of the cases^35^. Repolarization delays were also observed, however, they could not be significantly associated with arrhythmias (Figure 2)^_^35,38^_^. Cocaine is also highly arrhythmogenic, affecting the ion channels and thus the electrical stimulation of the heart. Cocaine inhibits sodium flow during depolarization, enhancing L-type calcium channels’ function and blocking the potassium channels (Figure 2)^39-42^. Cocaine consumption dramatically changes the electrocardiogram, shortening the PR interval, elongating the QTc interval, decreasing T-wave amplitude and increasing U-wave amplitude (Figure 2)^39,43^.

### 2.2. Cardiac heavy lifts: Hypertension

Hypertension is the disease of our century and the main cause of cardiomyopathy and heart failure. Caffeine abuse was believed to increase blood pressure and aggravate pre-existing cardiac conditions. However, the interaction between coffee consumption and high blood pressure was found only in patients with a genetic predisposition to essential hypertension^44^. Recent clinical trials did not associate caffeine consumption with significant elevation in blood pressure or related factors in other individuals with borderline hypertension^45^. Conversely, moderate caffeine intake was associated with a reduced risk of hypertension, by decreasing aortic stiffness and lowering the systolic and diastolic blood pressure^46^. A meta-analysis also showed a reduction in the risk of hypertension for each one cup/day increment of coffee consumption^47^.

Amphetamines and its derivatives increase blood pressure in a dose-dependent manner, through an αadrenergic -dependent mechanism^48^. Moreover, methamphetamine has the potential to induce pulmonary hypertension in individuals with specific polymorphisms in the methamphetamine-catabolizing enzyme carboxylesterase 1, by increasing reactive oxygen species, decreasing endothelial autophagy, and enhancing endothelial apoptosis^49^. However, the precise mechanisms of how amphetamines contribute to maintaining the increased blood pressure and pulmonary hypertension are still unclear. Similarly, methylphenidate administration showed an increased blood pressure in more than 12% of the medicated patients, particularly during the day-time^10^. Further studies are required to establish a definitive link between methylphenidate use and hypertension, along with investigating potential mechanisms, if a robust association is identified.

In contrast with other stimulants, cocaine has a demonstrated effect on inducing and maintaining hypertension. Cocaine abuse increases sympathetic effects on the cardiovascular system, causing enhanced inotropic and chronotropic effects^39^. Cocaine also induces acute hypertension by promoting increased vasoconstriction through elevated endothelin-1 levels^50^. The vasoconstrictive effects of cocaine extend to hindering the acetylcholine- and substance P-induced vasorelaxation, as well as nitric oxide release by blocking the action of the Ca2+-ATPase pump^51^, and Na+/K+ channels^52^.

### 2.3. Pipe predicament: Atherosclerosis

Atherosclerosis is the disease with the highest prevalence in the world population. It is the most common cause of myocardial infarction, the main cause of death worldwide. There is sufficient data associating substance abuse with the early and rapid progression of atherosclerosis processes, particularly in kids and young adults.

However, caffeine is excluded from this category, since there are multiple studies demonstrating its beneficial effects on vascular repair and regeneration, in experimental^53-55^and clinical trials^27,28^ that have statistical significance. Moderate caffeine intake, particularly in the range of 121 to 180 mg/day from coffee, is linked to lower risks of coronary artery disease. Acute caffeine ingestion significantly increased flow-mediated dilation and improved endothelial function, in both subjects with and without coronary artery disease, being associated with lower plasma markers of inflammation^56^. While the beneficial effect on coronary artery disease is clearly demonstrated, there is no data about the beneficial effect of caffeine consumption on peripheral vascular disease. The known positive effects are not related to atherosclerosis and it is mostly due to a competitive antagonist on adenosine receptors^57^. Caffeine, when administered acutely, was experimentally shown to decrease vasodilation induced by increased adenosine levels following hypoxia. These findings indicate that in the case of tissue hypoxia as a result of peripheral vascular disease, caffeine consumption could hinder the body’s vasodilation response^57^. Nevertheless, in patients suffering from claudication as a consequence of peripheral vascular disease, caffeine was shown to increase walking distance and maximal strength^58^.

**Figure 3:**
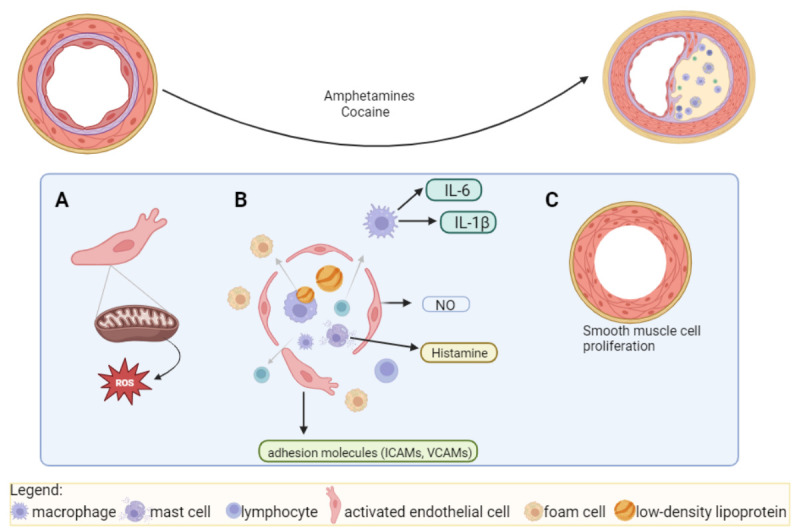
Effects of stimulants on induction and progression of atherosclerosis. (A) Increased production of reactive oxygen species. (B) Increased endothelial dysfunction, activation, permeability with increased macrophage/mast cells migration. (C) Smooth muscle cells proliferation.

Methamphetamine abuse is directly correlated with the risk and progression of atherosclerosis^59,60^. Methamphetamine enhances the production of reactive oxygen species^61-64^ in endothelial cells (Figure 3), and thus increasing the endothelial activation, permeability and proinflammatory gene expression (Figure 3), such as intercellular adhesion molecule-1, vascular cell adhesion molecule-1, and monocyte chemoattractant protein-1^59,60^. Methamphetamine-treated mice showed an increase in smooth muscle area, however, the observed elevated levels of interferon-γ and reduced transforming growth factor-β increased smooth muscle apoptosis, reducing extracellular matrix deposition, thus increasing the plaque vulnerability^59,60^ (Figure 3). Plaques from methamphetamine-treated mice also exhibit elevated proinflammatory T cells and macrophages. Treatment of human-derived macrophages with methamphetamine leads to the production of reactive oxygen species, interleukin-6, and interleukin-1β, known as pro-atherogenic factors^65^. Taken together, all current data highlight the increased risk and the rapid progression of atherosclerosis, following methamphetamine abuse in young adults, with increased risk for myocardial infarction at very young ages^30,66,67^.

Further, amphetamines may also cause premature peripheral arterial disease, by inducing premature atherosclerosis in the peripheral blood vessels^68-71^. The peripheral vascular manifestations are more severe in case of existent rheumatologic diseases^70^. However, many cases are caused by amphetamines’ peripheral vasoconstrictive actions, along with their vasculitis-inducing properties^69-71^. There have been reports of severe manifestations such as critical digital ischemia, that in some cases led to the development of gangrene, requiring amputation^70,71^. Methylphenidate abuse is currently not clearly associated with the atherosclerotic processes, both in coronary^72^ and peripheral artery disease^73^. However, peripheral vascular manifestations are more severe in case of coexisting rheumatic pathology^69,71,73^, evolving into gangrene and amputation in many cases^71^.

Cocaine users present the most severe forms of coronary atherosclerosis which leads to myocardial infarction in young individuals^74^. Current research highlights cocaine's inhibitory effect on nitric oxide release from endothelial cells, due mostly to increased endothelial disfunction^75^ (Figure 3). Furthermore, cocaine enhances the synthesis of cell adhesion molecules (e.g., ICAM-1, CD54, VCAM-1, ELAM-1), promoting the migration of low-density lipoprotein and leukocytes into the vessel wall. Between all leukocytes, cocaine affects particularly the mast cells^76^, increasing the release of proteolytic substances, increasing the endothelial permeability, as well as the uptake of low-density lipoprotein cholesterol by macrophages^77^ (Figure 3). Cocaine also increases the proliferation of intimal smooth muscle cells, intensifying the progression of atherosclerosis (Figure 3)^37,78^. Additionally, chronic cocaine abuse may weaken vessel walls, leading to apoptosis of vascular smooth muscle cells^79^ and increased plaque vulnerability and rupture. Cocaine abuse has been reported in association with some peripheral vascular manifestations, including peripheral vascular disease^80^, acute peripheral artery occlusive disease^81-83^ and secondary Raynaud’s phenomenon (although rare)^84,85^, manly by stimulation of the atherosclerotic process and peripheral vasoconstriction^81,86,87^. Nevertheless, the data regarding the relationship between cocaine abuse and peripheral vascular disease is quite scarce, but a study done on cocaine users revealed that they have poorer lower limb arterial perfusion compared to nonusers^80^.

### 2.4. Blocking the flow: Thrombosis and Thromboembolism

Thrombosis and thromboembolism are severe and multifactorial cardiovascular complications. There is a weak link between thrombosis, thromboembolism and stimulants abuse, however, the proof became more and more evident in favor of a direct correlation. Caffeine was not linked to the development of either thrombosis or embolism. It was revealed, though, that it was associated with enhanced formation of microparticles from the thrombocytes^88^ It is still debated whether or not caffeine offers antithrombotic protection, as some studies have indicated that caffeine inhibits platelet aggregation, while others have shown that this effect is actually due to other bioactive components that can be found in coffee together with caffeine^89^.

**Figure 4:**
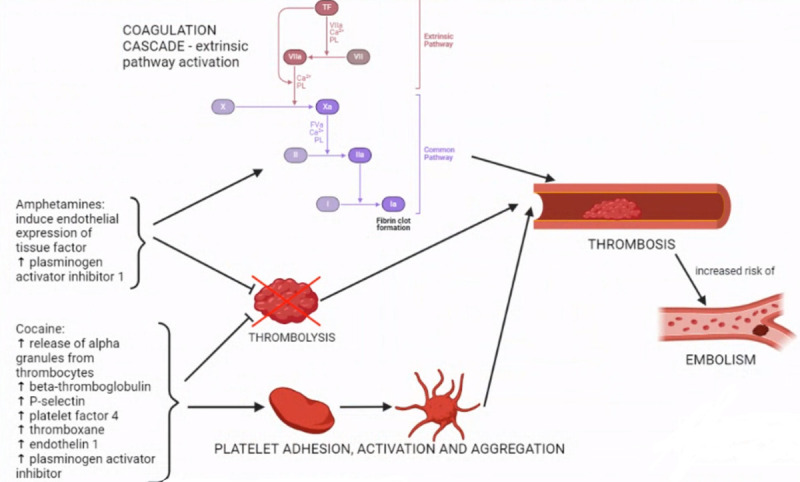
Thrombus formation and thromboembolism Amphetamines and cocaine influence coagulation pathways, platelet activation and aggregation, inhibiting thrombolysis, which leads to thrombus formation and vascular embolies.

Amphetamines abuse has been associated with increased thrombotic activity (Figure 4)^90^. They increase the expression of the well-known prothrombotic endothelial tissue factor in endothelial cells, by activating the D4 dopamine receptor, along with p38 and ERK (members of the mitogenactivated protein kinase family)^91^. Moreover, amphetamines were also found to impair fibrinolysis by enhancing levels of plasminogen activator inhibitor-1^92^. Thus, the use of amphetamines is associated with a higher risk of embolism^90^, particularly in patients with left ventricular assist device, pumps^93^, coronary diseases^94,95^, intracranial venous sinus thrombosis^96^ and orthopedic trauma^97^. Further, while methylphenidate use was not associated with thrombosis or embolus formation, cocaine is a well-known pro-thrombotic agent that activates all coagulation pathways^98-100^ (Figure 4). Cocaine use causes the overexpression of βthromboglobulin, P-selectin and platelet factor 4, leading to platelet activation and promoting the release of alpha-granules from thrombocytes^84,98^. Additionally, by increasing endothelin-1, Willebrand factor, fibrinogen and C reactive protein^100,101^, cocaine enhances endothelium activation and consequent leukocyte adhesion^99^. On top of these mechanisms, cocaine is also responsible for inhibiting the fibrinolytic pathways, as significantly elevated levels of plasminogen activator inhibitor-1 were reported following the administration of cocaine^90,100^. As localization, cocaine affect particularly deep veins^102^, aorta, renal vein and artery^98^, pulmonary artery, coronary arteries^100^, as well as small vessels of the colon and gallbladder^103^. These locations are predisposed to embolus development^82,86,99,104^ and acute peripheral occlusive disease, one of the most serious complications of peripheral vascular diseases^82,83,105^.

### 2.5. Oxygen strike: Ischemia and Myocardial Infarction

**Figure 5:**
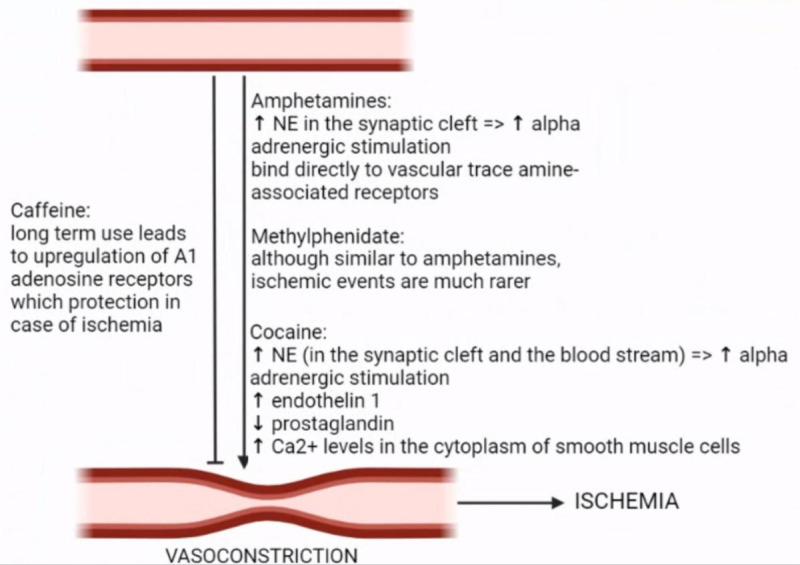
Ischemia and myocardial infarction Long-term consumption of caffeine protects tissue from ischemia, while amphetamines, methylphenidate and cocaine cause severe vasoconstriction through different mechanisms, leading altogether to ischemia.

Ischemia and infarction are extreme forms of disturbed tissue perfusion with dramatic consequences for all organs, particularly for the heart. Unfortunately, it is known that stimulant abuse leads to death, mostly due to myocardial ischemia and myocardial infarction. Caffeine was considered one major risk factor for tissue ischemia and infarction^106^ (Figure 5). However, it is still not clear if the effect is due to the caffeine itself or the associated bioactive components. Recent studies demonstrate the protective effect on the heart^27,28^ and brain^107^ during vascular obstructions, by antagonizing supposedly A1 and A2A adenosine receptors.

Amphetamines induce ischemia mainly by increasing the levels of monoamine neurotransmitters^108,109^, indirect sympathomimetic effects^110^, followed by vasoconstriction and organ ischemia^111^. The effects are not restricted to certain organs, but spread across multiple organ systems, such as the intestine (nonocclusive, located at an ileal level)^112^, pancreatic and biliary ducts^111^ and most severe, brain^113,114^ and myocardium^115,116^ (Figure 5). Besides myocardial infarction due to the coronary artery vasospasm, amphetamine abuse can affect the heart through increased thrombus formation and consecutive emboli^95^, as well as direct cardiotoxicity^115,117^, particularly by associated tobacco consumption^118^.

The methylphenidate use was associated with cerebral ischemia^69^ and peripheral ischemia manifested as secondary Raynaud’s phenomenon, acrocyanosis or livedo reticularis, due to the vasoconstrictive effect^70,71^ (Figure 5). However, there is no consistent data linking methylphenidate abuse with ischemic injury^119^. Emerging evidence pointing out a potentially elevated risk of myocardial infarction in children and young individuals with attention deficit hyperactivity disorder who undergo treatment with methylphenidate is still not concludent, and therefore, the risk-benefit balance should be meticulously considered, particularly in cases of mild forms of attention deficit hyperactivity disorder ^37,120^.

Cocaine-induced ischemia by several mechanisms that potentiate each other. Cocaine is capable of direct vasoconstriction, by affecting the calcium channels^81,121^ and stimulating the sarcoplasmic reticulum to release calcium into the cytoplasm of vascular smooth muscle cells^121^. Pharmacologically, cocaine is an indirect sympathomimetic substance, increasing the disponibility of norepinephrine and dopamine in synapses^81,122^, and from the medulla of the adrenal glands^81^. Additionally, cocaine affects the endothelial cells, releasing endothelin-1 (a powerful vasoconstrictor), and inhibiting the release of nitric oxide^81^. In thrombocytes, cocaine promotes an increase in thromboxane levels, a powerful vasoconstrictor^84^ (Figure 5). Once cocaine is metabolized, the vasoconstrictive effects are maintained by its metabolites, showing the extent and potential severity of this drug abuse^122^. In the heart, the effects of cocaine are dose-dependent^39^. Thus, at lower doses, cocaine induces an imbalance in oxygen demand and supply to the myocytes, consequently provoking localized ischemia^41^. Higher doses of cocaine exert inhibitory effects on sodium channels and impede norepinephrine uptake in ventricular myocytes, impeding ventricular contractility. Beside cardiac pathology^81,86,99,122-125^, the cocaine abuse associated with ischemic stroke^126,127^, ischemic colitis^121^, mesenteric ischemia^126^, glomerular ischemia^98^ and ischemia-based renal failure^127^.

### 2.6. Harmony disruption: Cardiomyopathy

Cardiomyopathies are the base of developing heart failure due to multiple factors, such as ischemia, and genetic variation, however, most of the inducers remain unknown. Caffeine abuse is not known to be related to any kind of cardiomyopathy. Amphetamines, instead, induce cardiomyopathies through different mechanisms, including elevated catecholamine levels, reactive oxygen species generation, accelerated apoptosis, heightened p53 activity, mitochondrial dysfunction^128^, metabolic dysregulation with fatty acid toxicity, coronary vasospasm, perfusion defects with associated myocardial ischemia^30^, altered gene expression, Cytochrome P450 polymorphism^129^, abnormal cardiac protein synthesis and function, along with disruptions in intracellular calcium homeostasis. Clinical studies demonstrated the gender-specific cardiac pathology of amphetamine abuse. While young males develop severe dilated cardiomyopathy, usually diagnosed at later stages with unfavorable prognosis, the female amphetamine users are diagnosed early due to Takotsubo-type presentation^130^.

Methylphenidate abuse also induced cardiomyopathies^131^, however, the underlying mechanism remains unclear. It is speculated that, besides sympathetic system stimulation^132^, the ultrastructural lesions induced directly by methylphenidate in the myocardial cells could lead in time to myocardial degradation and heart failure^133^. Cocaine abuse is well known as the cause of different types of cardiomyopathy by different mechanisms. When administered intranasally, cocaine stimulates central sympathetic outflow, affecting both cutaneous circulation, by vasoconstriction, promoting and heart, peripheral by inducing tachycardia^134^. This type of adrenergic stimulation can lead to Takotsubo cardiomyopathy, or brokenheart syndrome, which has been documented in cocaine abusers^135^. A study involving 430 patients with dilated cardiomyopathy revealed that cocaine consumers presented a significant increase in hypertrophic cardiomyocytes, fibrosis, apoptosis, necrosis^136^ and oxidative stress^137^, followed by deoxyribonucleic acid damages and myocardial dysfunction ^138^.

### 2.7. Confused wings: Valvulopathy

Valvulopathies are impairment of valvular function with perturbations in blood flow through the heart. Caffeine consumption seems to be associated with a reduced risk of developing or progressing heart valve disease in individuals with systolic blood pressure less than 160 mm Hg^139^. Contrary, amphetamines abuse increases heart valve fibroplasia by prolonged mitogenic responses in human valvular interstitial cells through the activation of 5-hydroxytryptamine 2B/serotonin serotonin receptors, increasing the valvulopathies incidence in these consumers^140^. In the case of methylphenidate abuse, there is no proof of any correlation or association with an increased risk of valvulopathies^141^. Evidence of cases of valvulopathy associated with cocaine abuse was identified in clinical studies with statistical significance, particularly in female consumers, however, the underlying mechanism remains unknown^142^.

### 2.8. Invasion unleashed: Vasculitis

Vasculitis represents a severe form of vascular damage by auto-immune reaction or infection. There is no association that caffeine will aggravate vascular damage during vasculitis. Amphetamine abuse, instead, has been reported as a clear cause of vasculitis^143-145^, more precisely been associated with the development of necrotizing angiitis^113,146^ in the nervous tissue^113,147^. The mechanism is not yet fully understood, however, it is speculated to be induced by either direct toxicity or a hypersensitivity reaction^147^. Methylphenidate has been reported as the causative agent behind cerebral vasculitis in several cases^148-150^. The mechanism behind the development of this pathology is unknown, but the structural and functional similarity between methylphenidate and drugs belonging to the amphetamine class is believed to be the reason behind this phenomenon^149,150^.

The vasculitis-inducing effects of cocaine are more pronounced after nasal administration, due to the effects of one of its adulterants – levamisole^151^. About 70% of cocaine is contaminated with levamisole^151^, since levamisole produces the same reaction as cocaine when cocaine purity is verified on the streets^152^. Cocaine use is associated with two main types of vasculitis, both positive for antineutrophil cytoplasmic antibodies^153^. Midline destructive lesions include the presence of caspases 3 and 9 (apoptotic markers), along with antimyeloperoxidase antibodies^153^, making it difficult to differentiate from granulomatosis with polyangiitis^151^. The second type of vasculitis is represented by necrotizing and leukocytoclastic vasculitis and thrombotic vasculopathy, along with retiform/stellar purpura – particularly on the cheeks, nose or ears. This type of vasculitis is speculated to be mainly induced by levamisole, and not cocaine^153-155^. One of the characteristics that differentiates this from primary anti-neutrophil cytoplasmic antibodyassociated vasculitis is the presence of anti-neutrophil cytoplasmic antibodies with dual specificity for both myeloperoxidase and proteinase 3 (PR3)^153^.

### 2.9. Brain maze: Cerebrovascular Disease

Cerebrovascular diseases include multiple pathologies affecting the adequate brain perfusion, such as ischemia or bleeding. Systemic hypertension is the most significant contributor to the increased risk of hemorrhagic stroke. A clinical study sought to explore the distinctions in cerebral blood flow before and after caffeine intake among individuals classified into low users, moderate users, and high users. Intriguingly, the results showed a decrease in cerebral blood flow in the gray matter, particularly in participants identified as low users^156^. Excessive use of caffeine was able to induce diffuse segmental vasoconstriction, as observed in the magnetic resonance brain imaging^157^.

Amphetamines abuse is well known to be associated with intracranial hemorrhages^158-160^ and subarachnoid hemorrhages^161,162^, due to systemic hypertension^163,164^. A forensic investigation has determined that post-mortem computed tomography findings, such as intracranial hemorrhages, midline shift, and hematoma volume, which are not associated with traumatic injury, should be regarded as suspicious features that could indicate intoxication with amphetamines^165^.

Further, both methamphetamine and cocaine contribute to vascular fatigue through their pharmacological actions, inducing hypertension and tachycardia. The more prolonged cardiovascular impact of methamphetamine could potentially explain the higher incidence of intracerebral hemorrhage associated with methamphetamine abuse compared to cocaine abuse^166^. Methamphetamine abuse was found to be associated also with ischemic stroke, lacunar^167^ or diffuse infarction^168^ by arterial stenosis, increased inflammation^160^ and accelerated atherosclerosis^169^. Cocaine abuse induces addiction by vasoconstriction of the prefrontal cortex, fostering compulsive cocaine intake^170^. Cocaine-induced cerebrovascular vasoconstriction leads, in severe cases, to ischemic stroke^171,172^. Nevertheless, cocaine abuse can trigger multiple cerebral pathologies, such as cerebral vasculitis, intracerebral hemorrhage, cerebral vasoconstriction and cerebrovascular spasm^173^.

### 2.10. Mindset makeover: Future Perspectives

The future perspectives in research on the cardiovascular effects of CNS stimulants are increasingly crucial as more individuals turn to these substances to cope with the demands of a stressful life. Understanding the long-term impact of these stimulants on cardiovascular health is essential, as current knowledge remains incomplete, particularly concerning individual variability and molecular mechanisms. Research must focus on developing preventive measures and educational campaigns to reduce reliance on stimulants. Additionally, innovative therapies are needed to help individuals overcome dependence and mitigate the adverse cardiovascular effects. As prevention is inherently more effective and less complex than treatment, prioritizing strategies to prevent stimulant misuse will be vital in safeguarding public health and minimizing the cardiovascular risks associated with these potent substances.

## 3. Conclusion

Our review provides a refined exploration of the cardiovascular effects induced by various stimulants, including caffeine, amphetamines, methylphenidate, and cocaine. These substances contribute to a spectrum of cardiovascular complications, from arrhythmias to atherosclerosis, thrombosis, ischemia and many more. While these stimulants present significant risks, the underlying mechanisms are incompletely investigated. By studying the responsible mechanism, we have challenged the prevailing myths, and canceled notions of widespread caffeine consumption as a major factor in increasing cardiovascular event risks. Now we know that caffeine demonstrates both protective and potentially detrimental effects on the cardiovascular system, highlighting the importance of moderation. Methylphenidate, commonly prescribed for attention deficit hyperactivity disorder, particularly in children and young adults, shows potential links to adverse cerebrovascular events and cardiomyopathy, requiring careful observation in clinical contexts and further investigation of the underlying molecular mechanisms. Considering individual and genetic variations, the multifaceted interactions between stimulants and cardiovascular health emphasize the necessity of personalized approaches. This review highlights that further research into inside molecular mechanisms is imperative. This not only enhances public education, medical treatment and healthcare policies, but also facilitates a personalized approach to mitigate morbidity and mortality associated with stimulants abuse. By doing so, we contribute to improving public cardiovascular well-being and promoting more effective strategies for intervention and care.
